# Protective and Recovery Effects of Resveratrol Supplementation on Exercise Performance and Muscle Damage following Acute Plyometric Exercise

**DOI:** 10.3390/nu13093217

**Published:** 2021-09-16

**Authors:** Chi-Chang Huang, Mon-Chien Lee, Chin-Shan Ho, Yi-Ju Hsu, Chien-Chang Ho, Nai-Wen Kan

**Affiliations:** 1 Graduate Institute of Sports Science, National Taiwan Sport University, Taoyuan 333325, Taiwan; john5523@ntsu.edu.tw (C.-C.H.); 1061304@ntsu.edu.tw (M.-C.L.); kilmur23@ntsu.edu.tw (C.-S.H.); ruby780202@ntsu.edu.tw (Y.-J.H.); 2 Graduate Institute of Metabolism and Obesity Sciences, Taipei Medical University, Taipei 11031, Taiwan; 3 Department of Physical Education, Fu Jen Catholic University, New Taipei City 24205, Taiwan; 093703@mail.fju.edu.tw; 4 Center for General Education, Taipei Medical University, Taipei 11031, Taiwan

**Keywords:** resveratrol, plyometric exercise-induced muscle damage (PEIMD), counter movement jump (CMJ), Wingate anaerobic test (WAnT), pressure pain threshold (PPT)

## Abstract

Plyometric exercise (PE) is an effective training method to increase muscle mass and strength. However, excessive or inappropriate conditions might cause exercise-induced muscle damage (EIMD). Resveratrol (RES) is a natural polyphenol plant antitoxin, which improves exercise performance, and exhibits anti-oxidation, anti-inflammatory, and anti-cancer effects. Therefore, this study investigated the effect of RES supplementation on the recovery of muscle damage, inflammation, soreness, muscle power, and anaerobic performance following plyometric-exercise-induced muscle damage (PEIMD). The present study was a double-blind, placebo-controlled research trial. Thirty-six young, untrained males were enrolled into the placebo (*n* = 12), RES-500 (500 mg RES/day, *n* = 12), or RES-1000 (1000 mg RES/day, *n* = 12) group by a jumping height-counterbalanced grouping design. At baseline, to pre-PEIMD, supplements were pre-loaded 7 days before they conducted PEIMD, and the exercise performance, delayed-onset muscle soreness (DOMS) and muscle damage biomarkers were measured over the experimental period at baseline, pre-PEIMD, and post-PEIMD at 2, 24, 48, and 72 h. As a result, we found that, at 72 h post-EIMD, the force peak (FP) and rate of force development (RFD) of the counter movement jump (CMJ) in RES groups showed no significant difference compared to that at baseline but was significantly greater than the placebo group. In the Wingate anaerobic test (WAnT), supplementation in the RES group had a better recovery effect on the relative peak power (RPP), relative mean power (RMP) and fatigue index (FI) (*p* < 0.05), especially in the high-dose group. For the detection of muscle pain after PEIMD, the RES supplement group was significantly better than the placebo group (*p* < 0.05). In addition, for muscle damage indexes, such as creatine kinase (CK) and lactate dehydrogenase (LDH), after PEIMD, supplementation with RES could significantly reduce and accelerate recovery (*p* < 0.05). In addition, the blood biochemical indicators of blood count, liver function, and kidney function showed that RES will not cause adverse risks to the human body. Our results suggest that replenishing RES in advance could effectively reduce muscle pain, increase exercise performance, and decrease muscle damage indicators caused by PEIMD, and the recovery was faster. Therefore, plyometric exercises combined with suitable RES supplementation could be an effective candidate for controlling muscle damage, improving physical adaption, and recovering anaerobic capacity.

## 1. Introduction

Plyometric exercise (PE) is a type of exercise based on the stretching and shortening cycle [1], which can be integrated into different types of muscle training, through muscle elastic energy and stretching reflex, to increase nervous system excitement and neuromuscular system response to effectively improve jumping, agility, muscle strength, muscle mass, and exercise performance [2,3]. Therefore, plyometric training is widely adopted by many coaches as one of the main training methods for athletes [4]. However, when unable to adapt or when overload training, it could cause damage to skeletal muscle fibers (i.e., exercise-induced muscle damage (EIMD)) [5,6], which could lead to the loss of calcium homeostasis, and produce inflammation and reactive oxygen species (ROS) to cause delayed onset muscle soreness (DOMS), which is characterized by stiffness, swelling of the exercise area, and decreased muscle function [3,7].

EIMD and DOMS usually occur from 12 to 24 h after exercise or training, and the maximum peak occurs between 24 and 72 h, before it gradually decreases [8]. This can be painful during palpation, contraction, or stretching of the affected muscle. The internal metabolic characteristics of the affected cells include leakage of skeletal muscle enzymes and proteins and increased levels of oxidative stress. The typical characteristics are elevated serum myoglobin (MB), LDH, and CK activity [9,10,11]. In addition, muscle damage could also lead to inflammation and oxidative stress, which reduces muscle function and strength [12]. As mentioned above, these adverse reactions after exercise will lead to a decline in muscle function and strength, especially in the athletes’ daily high-intensity training or tight competitions, which will reduce their performance in sport.

Resveratrol (trans-3,40,5-trihydroxystilbene (RES)) is a natural polyphenol found in many plant foods, such as grapes, red wine, apples, berries, peanuts, plums, and other oil seeds [13]. It has many pharmacological properties, including the prevention of metabolic syndrome [14] and cardiovascular disease [15], and can directly and indirectly reduce oxidative stress by neutralizing free radicals and upregulating endogenous antioxidant enzymes to induce an antioxidant effect [16]. In addition, RES downregulates NF-κB [17], inhibits the IGF-1R/Akt/Wnt pathway, and activates p53 expression [18], which has anti-inflammatory effects [19]. In previous studies, supplementation with RES has been shown to minimize the harmful effects of exercise-induced muscle damage during recovery [20]. In our previous research, we also found that RES supplementation could effectively improve muscle strength and endurance performance, and reduce fatigue biochemical parameters after exercise [21]. Additionally, resistance exercise training can accelerate the recovery of muscle strength and significantly improve muscle endurance performance [22]. In addition, after muscle contusion, supplementation with RES can effectively reduce damage indicators, such as CK and LDH, and promoted the regeneration of muscle satellite cells [23]. However, these experiments were conducted through animal models, and further experiments are needed to determine the benefits for human muscle recovery.

In this study, we integrate previous related studies to explore the prevention and protection benefits of different doses of RES supplementation on non-athletic males for plyometric-exercise-induced muscle damage (PEIMD) through the evaluation of exercise performance, muscle injury indicators, inflammation, and soreness.

## 2. Materials and Methods

### 2.1. Experimental Design

Trans-resveratrol samples were purchased from Biotivia (Biotivia Inc., New York, NY, USA). Each capsule contains trans-resveratrol (>98%, 500 mg), piperine (95%, 5 mg), and ploydation (98%, 5 mg). The supplement capsules were separated into two equal parts taken in the morning and evening each day for a total of 10 days following baseline assessments.

**Figure 1:**
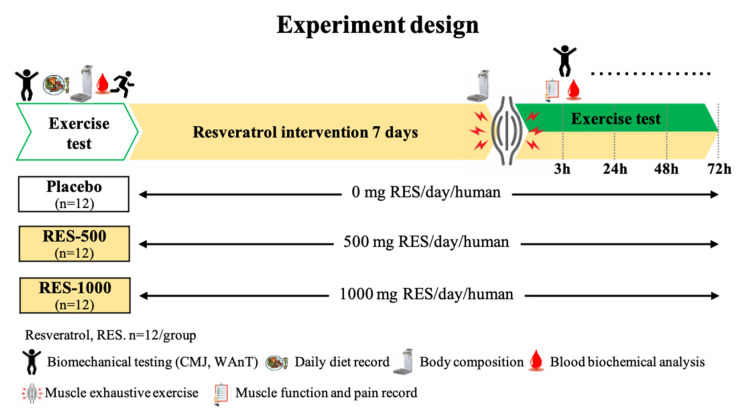
Experimental design overview: participants were supplemented for 10 days with placebo, 500 mg/day resveratrol (RES-500), or 1000 mg/day resveratrol (RES-1000), and an acute plyometric-exercise-induced muscle damage (PEIMD) protocol was conducted pre-PEIMD (day 7).

This study was a double-blind, placebo-controlled research trial. Thirty-six participants were enrolled, counterbalanced by their performance in the countermovement jump (CMJ) test, and divided into three groups: placebo (1000 mg/day methylcellulose), RES-500 (500 mg/day RES + 500 mg/day methylcellulose), or RES-1000 (1000 mg/day RES), respectively. Participants visited the laboratory at least six times. Upon their first visit, medical histories and exercise habits were recorded, and anthropometry, exercise performance, and muscle soreness were measured 10 days before placebo or resveratrol supplementation. After PEIMD, the exercise performance, muscle soreness, and muscle damage biomarkers were measured for 72 h to track conditions of muscle recovery (Figure 1). Furthermore, participants were instructed to follow their normal diet throughout the duration of the study and asked to record their dietary intake for analysis.

### 2.2. Participants

In this study, participants were excluded if they had any musculoskeletal or metabolic disorders, including neuromuscular/neurological diseases, heart/cardiopulmonary diseases, diabetes, thyroid disease, hypogonadism, hepatorenal disease, autoimmune diseases, or cancer. Furthermore, all recruited individuals refrained from consuming any supplements or medication, such as non-steroidal anti-inflammatory or steroidal drugs, for four weeks prior to participation in this study and throughout the entire experimental period. Alcohol or caffeine consumption, cryotherapy or massage treatments, and resistance or high-intensity aerobic exercise were not allowed 48 h prior to testing, throughout the entire testing period, and 12 h prior to each visit. The study was conducted according to the guidelines of the Declaration of Helsinki, and approved by the Joint Institutional Review Board of Taipei Medical University (TMU-JIRB, no. N20170250, Taipei, Taiwan). Participants were made aware of all risks and benefits of the procedures and signed the informed consent in person. Thirty-six young, non-athletic males (age: 21.09 ± 1.33 years old; height: 173.17 ± 6.19 cm; and body mass: 69.92 ± 7.78 kg) were recruited for the study.

### 2.3. Anthropometric Measurements

An InBody 570 device (In-body, Seoul, South Korea), which is a bioelectrical impedance analyzer (BIA), with a within-60 s multi-frequency principle of 1, 5, 50, 260, 500, and 1000 kHz, was used to measure body composition. To perform the measurements, the subjects stood on the footing electrodes and held the sensing handles with two hands. During the measurements, the subjects kept their arms open, which left their trunk at a 30° angle, and did not speak or move. All subjects were asked to fast for at least 8 h before the test [24].

### 2.4. Plyometric-Exercise-Induced Muscle Damage (PEIMD)

In this study, we utilized one kind of plyometric exercise protocol to induce muscle damage. All participants performed 10 sets of 10 maximal vertical jumps, interspersed with a one-minute recovery between each set. Prior to starting the exercise, a maximal vertical jump was performed. The participants were instructed to perform each jump with maximal effort to reach the target height with a colorful taping mark made by the fingertips at the highest point of the jump, and the authors encouraged the participants to reach the mark. On landing, participants were asked to adopt a knee joint angle of 90°. This protocol has been used successfully to induce PEIMD in our previous studies [25].

### 2.5. The Countermovement Jump (CMJ) Test

In this study, the CMJ was used to evaluate the lower limb maximum strength, explosive force, and speed [26]. Participants wore a reflective marker on the middle fingertip of their dominant hand and stood with both feet on a force plate. They were asked to squat down until their knees bent to 90° and then to immediately jump as high as possible. A motion capture system with 10 high-speed cameras (VICON T40, Oxford Metrics Ltd., Oxford, UK) and a force plate (9281B; Kistler Ltd., Winterthur, Switzerland) were used synchronously. The sampling rates of the motion capture system and force plate were set at 200 and 1000 Hz, respectively. Each participant repeated the test three times, the average value was taken as a calculation parameter, and the individual weight was used to standardize body weight (BW). The RFD, FP, and VJH were calculated using the self-developed MATLAB (R2017b, MathWorks, Natick, MA, USA) program [27].

### 2.6. Wingate Anaerobic Test (WAnT)

After a standard warm-up, all subjects were assessed with the classical WAnT on a cycloergometer (Monark 894E, Varberg, Sweden) in a 30 s “go all out” ultramax test. The seat height was adjusted to the satisfaction of each participant, and toe clips prevented the feet from slipping off the pedals. Before the initial test, the subjects warmed up for 5 min, and the power was approximately 50 W. After the warm-up, two preparation exercises lasting 3 s, during which the actual test load was 3% of their own body weight, were applied to accustom the participant to resistance [28]. The test started, and the resistance was set on the friction belt of the dynamometer. External loading was estimated individually at 5% body weight. The recorded results were the relative mean power (W/kg), relative peak power (W/kg), and fatigue index (%). This assessment was performed before and 24 and 72 h after the exhaustive exercise program.

### 2.7. Muscle Soreness

**Figure 2:**
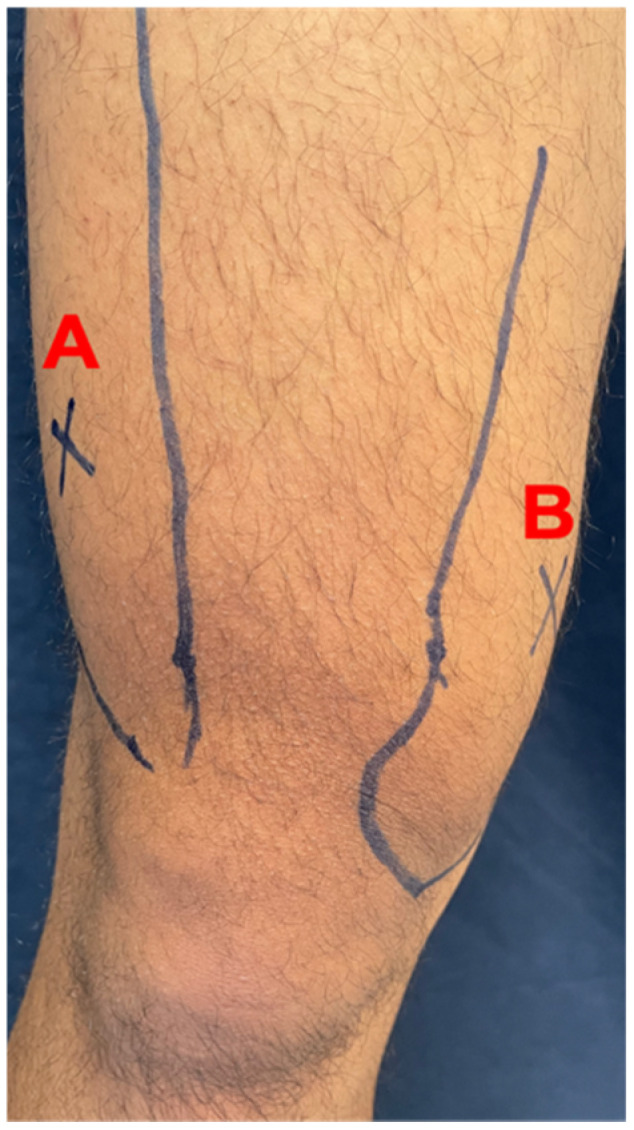
Measurement points in anterior thigh muscles. (**A**) vastus lateralis, (**B**)vastus medialis. The photo provided by Dr. Mon-Chien Lee.

In this study, values of muscle soreness were presented by the pain threshold to determine the minimum pressure that triggers pain at the point of the target tissue. PPT diagnostic testing was performed with the advantages of a 3i digital force/torque indicator (Mark-10, New York, NY, USA). Pressure algometry is a reliable measure of pain in muscle, joints, tendons, and ligaments [29]. For muscle soreness evaluation, the lower parts of the vastus lateralis (Figure 2A) and vastus medialis (Figure 2B) were used.

### 2.8. Clinical Biochemistry, Hematology, and Inflammation Cytokines Analysis

Venous blood was collected at baseline, pre-PEIMD, and 2, 24, 48, and 72 h post-PEIMD for biomarker assessments, including muscle damage, liver and renal functions, and inflammation responses. As indirect markers of muscle damage, plasma concentrations of CK and LDH were determined using an automatic analyzer (Hitachi 7060, Hitachi, Tokyo, Japan). To monitor the influence of liver and renal functions over the course of the experimental period, clinical biochemical variables, namely AST, ALT, BUN, and CREA, were analyzed following a short-term RES supplementation and an acute polymetric exercise. These clinical biochemical parameters were determined by an automatic analyzer (Hitachi 7060, Hitachi, Tokyo, Japan). The CBC profiles were also analyzed at 2 and 24 h post-PEIMD (MindrayBC-2800 Vet, Shenzhen, China).

### 2.9. Statistical Analysis

Major data are expressed as mean ± SEM. Statistical analyses were performed by SAS 9.0 (SAS Inst., Cary, NC, USA). Between- and within-group differences (at different time points) were analyzed by one-way analysis of variance (ANOVA). By using the IBM SPSS Statistics ver. 24.1 (IBM Co., Armonk, NY, USA), repeated-measure analysis of variance was used to compare RES and placebo at specified time points during recovery. A paired *t*-test with Bonferroni adjustment was used to compare treatment differences at each time point with pre-PEIMD. Differences were considered statistically significant at *p* < 0.05. We used G*power to calculate the effect size [30]. According to the calculation, the effect size f was 0.6994612. After calculating the sample size based on effect size f, α error problem of 0.05, and power of 0.95, we found that the total effective sample size in this experiment is 36. Therefore, 12 subjects in each group are in line with the statistical power.

## 3. Results

### 3.1. Subject Characteristics

Characteristics of participants are presented as Supplementary Material (Table S1). There were no significant differences in table characteristics, including body mass index (BMI), muscle mass, and fat percentage, at baseline among groups. Furthermore, no significant differences in carbohydrate intake (g/day), protein intake (g/day), or total calories consumed (kcal/day) were found at baseline among groups.

### 3.2. Effect of RES Supplementation on Vertical Jump Height of Exercise Performance

**Figure 3:**
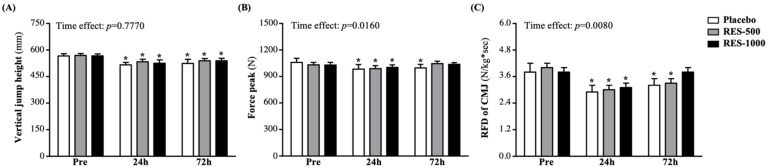
Effect of supplementation with RES on (**A**) vertical jump height, (**B**) force peak, and (**C**) RFD of CMJ. Data are presented by mean ± SEM. Administration effects were statistically analyzed with a paired Student’s *t*-test, * *p* < 0.05. RFD, rate of force development; CMJ, countermovement jump.

Vertical jump height (VJH) is a popular test for evaluating explosive or muscle strength performance. Compared with pre-PEIMD, VJH levels in the placebo, RES-500, and RES-1000 groups at 24 h post-PEIMD significantly decreased by 8.9% (*p* < 0.0001), 6.35% (*p* = 0.0001), and 7.48% (*p* = 0.0014), respectively, and at 72 h post-PEIMD were significantly lower by 7.4% (*p* = 0.0016), 5.4% (*p* = 0.0001), and 5.1% (*p* = 0.0002), respectively (Figure 3A). However, vertical jump performance showed no interaction effects. No significant time effect has been shown (*p* = 0.7770).

The force peak (FP) is measured by a force platform from the subject performing the max countermovement vertical jump. At pre-PEIMD, the FP did not differ among the placebo, RES-500, and RES-1000 groups (Figure 3B). At 24 h post-PEIMD, the FP in the placebo, RES-500, and RES-1000 groups was significantly lower than pre-PEIMD by 7.2% (*p* = 0.008), 4.1% (*p* = 0.0008), and 2.6% (*p* = 0.0008), respectively. At 72 h post-PEIMD, the FP in the placebo group was significantly lower than pre-PEIMD, by 5.9% (*p* = 0.0259), but the RES-500 and RES-1000 groups showed no significant difference in comparison with pre-PEIMD. A significant time effect has been shown (*p* = 0.0160).

The RFD is measured by a force platform from the subject performing the max countermovement vertical jump. At pre-PEIMD, there was no significant difference among the placebo, RES-500, and RES-1000 groups (Figure 3C). At 24 h post-PEIMD, the placebo, RES-500, and RES-1000 groups showed significantly lower RFD, by 23.7% (*p* = 0.0000), 26.0% (*p* = 0.0000), and 17.2% (*p* = 0.0007), respectively, compared with pre-PEIMD. At 72 h post-PEIMD, RFD in the placebo and RES-500 groups were significantly lower than pre-PEIMD by 16.0% (*p* = 0.0064) and 17.4% (*p* = 0.0000), respectively, but RES-1000 showed no significant difference. A significant time effect has been shown (*p* = 0.0080).

### 3.3. Effect of RES Supplementation on Wingate Anaerobic Test (WAnT) of Exercise Performance

**Figure 4:**
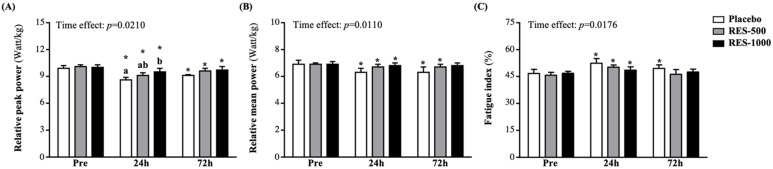
Effect of supplementation with RES on (**A**) relative peak power (RPP), (**B**) relative mean power (RMP), and (**C**) fatigue index. Data are presented by mean ± SEM. Different superscript letters (a, b) indicate significant differences among the groups at *p* < 0.05, and administration effects were statistically analyzed with a paired Student’s *t*-test, * *p* < 0.05.

At pre-PEIMD of RPP, there were no significant differences among the groups. Although the placebo, RES-500, and RES-1000 groups showed significant decreases of 13.2% (*p* < 0.0001), 10.0% (*p* < 0.0000), and 4.3% (*p* = 0.0007), respectively, at 24 h post-PEIMD compared to pre-PEIMD, the RES-1000 group showed significantly higher results than the placebo group by 1.11-fold (*p* = 0.0378). At 72 h post-PEIMD, the placebo, RES-500, and RES-1000 groups showed significantly lower results than pre-PEIMD, by 8.1% (*p* = 0.0038), 4.7% (*p* = 0.0014), and 2.9% (*p* = 0.0009), respectively (Figure 4A). A significant time effect has been shown (*p* = 0.0210).

The RMP in the placebo, RES-500, and RES-1000 groups was not significantly different at pre-PEIMD. However, the placebo, RES-500, and RES-1000 groups showed significantly lower results than pre-PEIMD by 8.9% (*p* = 0.0041), 3.3% (*p* = 0.0105), and 2.5% (*p* = 0.0061), respectively, at 24 h post-PEIMD. At 72 h post-PEIMD, the placebo and RES-1000 groups were significantly lower by 8.5% (*p* = 0.0474) and 2.7% (p = 0.0369) compared with pre-PEIMD (Figure 4B). A significant time effect has been shown (*p* = 0.0110).

As shown in Figure 4C, no significant difference in FI among the placebo, RES-500, and RES-1000 groups at pre-PEIMD was observed. However, the placebo and RES-500 groups at 24 h post-PEIMD showed significantly lower results than at baseline, by 12.1% (*p* = 0.0003) and 9.8% (*p* = 0.0002). At 72 h post-PEIMD, only the placebo group showed significantly lower results than pre-PEIMD, by 6.1% (*p* = 0.0021). A significant time effect has been shown (*p* = 0.0176).

### 3.4. Effect of RES Supplementation on Pressure Pain Thresholds (PPT)

**Figure 5:**
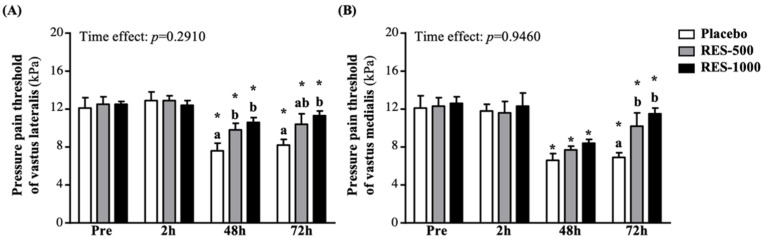
Effect of supplementation with RES on pressure pain threshold of (**A**) vastus lateralis and (**B**) vastus medialis. Data are presented by mean ± SEM. Different superscript letters (a, b) indicate significant differences among the groups at *p* < 0.05, and administration effects were statistically analyzed with a paired Student’s *t*-test, * *p* < 0.05.

In this study, we used pressure algometry for pain threshold testing at the measurement points of the anterior thigh muscles at 24 and 72 h post-EIMD, which is appropriate for determining the minimum pressure that triggers pain at two points in the anterior thigh muscles (e.g., vastus lateralis and vastus medialis). The pressure pain threshold of vastus lateralis in the RES-500 and RES-1000 groups at 24 h post-PEIMD was significantly increased compared to the placebo group by 1.30- (*p* = 0.0294) and 1.41-fold (*p* = 0.0042), respectively, but at 72 h post-PEIMD, only the RES-1000 group showed a significantly higher threshold compared to the placebo groups, by 1.38-fold (*p* = 0.0071). In addition, compared with pre-PEIMD, the placebo, RES-500, and RES-1000 groups showed significantly lower thresholds, by 37.3% (*p* = 0.0000), 21.4% (*p* = 0.0000), and 14.6% (*p* = 0.0000), respectively, at 24 h post-PEIMD, and by 32.1% (*p* = 0.0002), 17.2% (*p* = 0.0001), and 9.4% (*p* = 0.0022), respectively, at 72 h post-PEIMD (Figure 5A). No significant time effect has been shown (*p* = 0.2910).

As shown in Figure 5B, the pressure pain threshold of vastus medialis in the RES-500 and RES-1000 groups was significantly increased at 72 h post-PEIMD compared to the placebo group by 1.48- (*p* = 0.0142) and 1.68-fold (*p* = 0.0009). In addition, compared with pre-PEIMD, the placebo, RES-500, and RES-1000 groups showed significantly lower thresholds, by 45.2% (*p* = 0.0002), 37.2% (*p* < 0.0001), and 33.6% (*p* < 0.0001), respectively, at 24 h post-PEIMD; and by 42.9% (*p* = 0.0003), 17.3% (*p* = 0.0173), and 8.6% (*p* = 0.0294), respectively, at 72 h post-PEIMD. No significant time effect has been shown (*p* = 0.9460).

### 3.5. Effect of RES Supplementation on Muscle Damage Biomarkers

**Figure 6:**
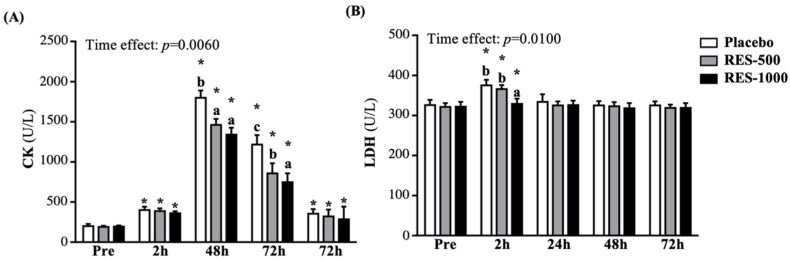
Effect of supplementation with RES on (**A**) CK and (**B**) LDH. Data are presented by mean ± SEM. Different superscript letters (a, b, c) indicate significant differences among the groups at *p* < 0.05, and administration effects were statistically analyzed with a paired Student’s *t*-test, * *p* < 0.05. CK, creatine kinase; LDH, lactate dehydrogenase.

In the current study, all groups showed significantly increased creatine kinase (CK) activity at 2, 24, 48, and 72 h post-PEIMD, compared with pre-PEIMD (*p* < 0.05), respectively; it reached its peak at 24 h post-PEIMD, then decreased until 72 h post-PEIMD. However, compared with the placebo group, the CK activity in the RES-500 and RES-1000 groups was significantly decreased at 24 h post-PEIMD, by 18.73% (*p* = 0.0081) and 25.53% (*p* = 0.0005), respectively, and at 48 h post-EIMD, by 29.54% (*p* < 0.0001) and 38.51% (*p* < 0.0001), respectively. However, there was no significant difference among the placebo, RES-500, and RES-1000 groups at 72 h post-PEIMD (Figure 6A). A significant time effect has been shown (*p* = 0.0060).

As shown in Figure 6B, all groups showed significantly increased lactate dehydrogenase (LDH) activity at 2 h post-PEIMD compared to pre-PEIMD (*p* < 0.05), but recovery at 24 h post-EIMD showed no differences. At 2 h post-PEIMD of LDH activity, only the RES-1000 group showed a significant decrease, by 12.38% (*p* = 0.0131), compared to the placebo group. A significant time effect has been shown (*p* = 0.0100).

### 3.6. Effect of RES Supplementation on Blood Biochemistry Markers

The blood biochemistry markers are presented as Supplementary Material (Table S2). Liver function biomarkers, including aspartate aminotransferase (AST) and alanine aminotransferase (ALT), did not differ among groups (*p* > 0.05). In the current study, all values for liver functions were in the normal range regarding AST (13–39 mg/dL) and ALT (7–52 mg/dL).

Renal function biomarkers, including blood urea nitrogen (BUN) and creatine (CREA), did not differ among groups (*p* > 0.05). In the current study, all values for renal functions were in the normal range regarding BUN (7–25 mg/dL) and CREA (0.6–1.3 mg/dL).

### 3.7. Effect of RES Supplementation on Complete Blood Count (CBC) Profiles

All blood count profiles for the current study are shown in Table 1. There was no significant difference in the data of all items among the groups. However, regardless of the group, the neutrophils, lymphocytes, and neutrophil/lymphocyte ratio (NLR) at 24 h post-PEIMD were significantly decreased compared to pre-PEIMD (*p* < 0.05).

RBC profiles did not differ among groups (*p* > 0.05) at pre-PEMID, 2 h post-PEIMD, and 24 h post-PEIMD (data not shown). Values of RBC profiles at pre-PEIMD compared to those at 24 h post-PEIMD among groups showed no significant difference (*p* > 0.05).

## 4. Discussion

In this study, the commonly used PE-induced PEIMD model was applied [31], and different doses of RES were supplemented from 1 week before PEIMD to 72 h post-PEIMD. Our research results showed that although at 24 h post-PEIMD, decreased strength performance, as well as increased fatigue index, muscle pain, and biomarkers of muscle damage in the blood, was observed, this gradually recovered at 72 h post-PEIMD. However, RES supplementation could significantly prevent, delay, and reduce the negative effects of muscle damage, and has the benefit of accelerating recovery, especially in the high-dose group.

In strength and physical fitness research, vertical jumping is used to evaluate athletes’ lower limbs, including speed, maximum strength, agility, explosive power, and anaerobic performance [32,33]. At present, there are still many different views on the impact and relevance of EIMD to CMJ. Past studies have pointed out that after EIMD, the height of CMJ decreases by an average of 9% [34], and could be gradually recovered within 24–48 h through nutritional supplements or massage methods [35,36]. However, another study negatively speculated that due to fatigue accumulation, CMJ height might decrease or not change significantly [37]. A recent study confirmed this phenomenon, in which the dynamic parameters during the CMJ protocol are highly dependent on the athlete’s training background (i.e., anaerobic and endurance athletes) and independent of jump performance [38]. Nevertheless, in functional and performance tests, explosive power may be more important than maximum jump performance. Among them, maximum RFD and FP are often used as important indicators to assess muscle strength and explosive power [39]. In our previous study, we also found that supplementation with lemon verbena extract for 10 days has no significant effect on the vertical jump height, but seems to be better able to maintain explosive power and other strength performance measures [25]. In our results, the strength performance was significantly reduced at 24 h post-PEIMD. Although supplementation with RES had no substantial effect on VJH (Figure 3A), it seemed to have a significant effect on the recovery and improvement of explosive power (Figure 3B,C and Figure 4).

In addition to the vertical jumping ability test, many previous studies have also used the Wingate anaerobic test to evaluate the effects of lower limb muscle strength and anaerobic power after PEIMD [40], and it has been proved to correlate with the CMJ test [41]. Our research seemed to confirm this result. At 24 h post-PEIMD, the RPP and RMP of all groups were significantly lower than pre-PEIMD. However, compared with the placebo group, supplementation with RES showed significantly better protection mechanisms and recovery benefits (Figure 4A,B). These showed the same trends as the results of the vertical jump. In addition to evaluating strength performance, WAnT can also be used as a reference for exploring energy use and FI. In a 30 s full sprint of WAnT, approximately 75% of the energy requirement is provided by anaerobic metabolism [42]. In the first 6 s, the maximum peak power output could be generated in a short time due to the need to reserve energy, such as free adenosine triphosphate (ATP) and phosphocreatine (PCr). However, after PCr storage was significantly depleted, this compromised power output coincided with the time at which glycolysis attained its maximum rates [43]. RES is confirmed to activate the expression and biological activity of SIRT1. By directly activating FOXO1, glucose metabolism was converted into gluconeogenesis, and glucose release was promoted [44]. In addition, in our previous animal study, it was also found that RES could provide the energy required for exercise and accelerated the recovery of fatigue after exercise [21]. Therefore, in the current study, supplementation with RES could reduce the increase in FI after PEIMD by the WAnT test in humans and accelerate its recovery (Figure 4C). We infer that it may be the effect of the above mechanism, but the specific effect needs to be further confirmed.

PPT is a subjective sensation, but an objective quantitative evaluation method is used to quantify the pain with the least stress. Past research has confirmed the effectiveness of PPT as an evaluation tool; however, the measuring point and the operator should be as consistent as possible to avoid accuracy errors caused by active intervention [45]. Previous studies have shown that PEIMD might be caused by the initial mechanical pressure-induced micro-tearing of myofibrils, leading to inflammation and the release of harmful stimuli (i.e., bradykinin and nerve growth factor). In addition, a large influx of neutrophils produced by the initial injury would stimulate lipid peroxidation, leading to an increase in free radicals, inflammatory molecules, and H^+^. As a result, muscle nociceptors are more sensitive, causing soreness and pain [46]. Due to the association between pain and oxidative stress and inflammation, taking antioxidant-rich supplements after muscle injury exercise could reduce inflammation and relieve muscle pain. RES has potential antioxidant and anti-inflammatory properties and has been proven to minimize the effect of EIMD on soreness and pain [47]. In the past, many experiments related to EIMD or inflammation also tested CBC, and used neutrophils and eosinophils and so on as evaluation indicators [48]. However, in the past studies related to resveratrol, no improved benefits have been found [49]. In our results, PEIMD significantly reduced neutrophils after 24 h compared to 2 h post-PEIMD, but RES supplementation did not seem to have a significant effect (Table 1). However, in the results of PPT, both the vastus lateralis and vastus medialis had the most severe pain at 24 h post-PEIMD and the pain was gradually relieved at 72 h post-PEIMD. Supplementation with RES could significantly reduce and accelerate the recovery of muscle pain (Figure 5A,B).

For sports medicine and physiology, strenuous exercise could cause muscle injury, in which case cell membrane integrity would be compromised, and cytosolic enzymes would leak into the serum [50]. Serum CK is an important clinical biomarker of muscle injury, such as severe muscle breakdown, muscular dystrophy, autoimmune myositis myocardial infarction, and acute renal failure [21]. Therefore, the appearance of CK in the blood is usually known as an indirect sign of muscle damage. The concentration of CK begins to increase within 2–12 h after the occurrence of muscle injury, reaches a maximum within 24–72 h, and decreases as the muscle injury subsides within 2–5 days [51]. In addition, LDH, myoglobin, AST, ALT, and so on are all muscle damage biomarkers [52]. Previous studies have shown that supplementation with RES could reduce LDH, CK, 8-hydroxy-2′-deoxyguanosine (8-OHdG), malondialdehyde (MDA), and 4-hydroxy-2-nonenal (4-HNE), and prevent oxidative damage and lipid peroxidation caused by strenuous exercise [53]. We previously confirmed in an animal experiment that supplementation with RES could effectively improve exercise performance and reduce fatigue biochemical values, such as CK, after exercise [21]. The current study also confirmed that supplementation with RES in humans could significantly reduce CK and LDH after PEIMD and accelerate recovery (Figure 6A,B).

Based on the above results and literature discussion, we found that although many studies have shown that resveratrol supplementation has the benefits of protecting oxidative damage and lipid peroxidation, the digestion, absorption, and metabolism of exogenous resveratrol may vary from person to person. It is affected by many factors, including body weight, enzymes activity, metabolic rate, and co-consumption of nutrients [47]. However, in the current study, supplementation with RES on humans, especially a large dose of 1000 mg/day, could effectively reduce the pain, the decline in strength performance, the increase in blood muscle damage, and the inflammation caused by PEIMD, and could accelerate recovery. In addition, high doses of RES would not cause harm or burden on liver and kidney function.

A previous study had shown that the content of resveratrol in natural sources includes grapes (contain 0.16–3.54 µg/g), red wine (contain 0.1–14.3 µg/mL), blue berries (contain ~0.016 µg/g), peanuts (contain 0.02–1.92 µg/g), and *Fallopia japonica*, Japanese knotweed (contains 524 µg/g) [54]. If we want to supplement at least 500 mg or more, it may cause a greater burden on the body due to excessive food intake. In addition, after resveratrol was ingested 77–80%, it was absorbed in the intestine [47]. Therefore, when only evaluating plasma, it peaks 30 to 60 min after being supplemented with resveratrol [55]. In addition, up to 76% of resveratrol is not accounted for when only plasma is evaluated [56]. RES metabolites are absorbed by active transport and in the blood stream. The free form of resveratrol is bound to albumin and lipoproteins. When these complexes reach cells having albumin and lipoprotein receptors, RES dissociates and is rendered free to enter cells. Hence, these complexes act as reservoirs for RES and help in its distribution [57]. It is well known that RES has anti-oxidant and anti-inflammatory functions. In this study, we aimed to explore the direct functional benefits of RES supplementation on muscle and physical performance at 2, 24, 48, and 72 h after an acute plyometric exercise. Thus, we chose to look at the more relative clinical biochemistry of blood than the classic antioxidant markers.

This study had several limitations. First, the study focused on healthy adults who were not athletes, and only males were included, which limited the generality of these results to other populations. Second, muscle puncture is among the more direct and accurate assessment methods. However, we had not received medical professional training. In addition, it is difficult to obtain IRB approval. Third, we could not provide subjects with a fixed diet to standardize nutrition. We could only ask the subjects to record their daily diet as much as possible, which was then analyzed and evaluated by a professional nutritionist. Data are presented as Supplementary Material (Table S1).

## 5. Conclusions

In sum, our results suggest that preloading RES supplements can reduce the levels of muscle damage, inflammation, and soreness caused by PEIMD, and subsequently affect the recovery of power and anaerobic performances. This might be a potential strategy to maintain the quality and intensity of training with the high repetition demand of eccentric exercises. Therefore, plyometric exercises combined with suitable RES supplementation could be an effective candidate for controlling muscle damage, improving physical adaption, and recovering anaerobic capacity.

## Figures and Tables

**Table 1:** The effect of RES supplementation on the blood count profiles of subjects.

Parameters	Group	Pre	2 h	24 h
WBC (cells/mcL)	Placebo	6433 ± 239 ^a^	6887 ± 334 ^a^	5723 ± 284 ^a^
	RES-500	6518 ± 289 ^a^	6945 ± 413 ^a^	5775 ± 350 ^a^
	RES-1000	6597 ± 427 ^a^	6943 ± 546 ^a^	5683 ± 491 ^a^
Neutrophils (%)	Placebo	69.6 ± 3.8 ^a^	65.5 ± 2.4 ^a^	54.2 ± 1.6 ^a,^*
	RES-500	69.7 ± 3.8 ^a^	64.6 ± 1.2 ^a^	54.6 ± 2.2 ^a,^*
	RES-1000	69.8 ± 2.4 ^a^	65.1 ± 1.6 ^a^	54.5 ± 2.3 ^a,^*
Lymphocytes (%)	Placebo	25.3 ± 1.7 ^a^	26.0 ± 2.0 ^a^	36.4 ± 1.5 ^a,^*
	RES-500	24.9 ± 2.9 ^a^	26.7 ± 1.1 ^a^	36.1 ± 2.2 ^a,^*
	RES-1000	25.9 ± 1.8 ^a^	26.3 ± 1.4 ^a^	36.1 ± 2.2 ^a,^*
Monocytes (%)	Placebo	4.3 ± 0.8 ^a^	5.0 ± 0.3 ^a^	5.4 ± 0.1 ^a^
	RES-500	4.1 ± 0.8 ^a^	5.4 ± 0.4 ^a^	5.4 ± 0.4 ^a^
	RES-1000	4.8 ± 0.8 ^a^	5.6 ± 0.3 ^a^	5.6 ± 0.3 ^a^
Eosinophils (%)	Placebo	0.8 ± 0.2 ^a^	0.7 ± 0.1 ^a^	0.8 ± 0.1 ^a^
	RES-500	0.8 ± 0.2 ^a^	0.7 ± 0.1 ^a^	0.8 ± 0.1 ^a^
	RES-1000	0.8 ± 0.3 ^a^	0.6 ± 0.1 ^a^	0.7 ± 0.1 ^a^
Basophils (%)	Placebo	2.1 ± 0.6 ^a^	2.7 ± 0.3 ^a^	1.5 ± 0.1 ^a^
	RES-500	2.5 ± 0.4 ^a^	2.5 ± 0.2 ^a^	1.6 ± 0.2 ^a^
	RES-1000	2.4 ± 0.3 ^a^	2.6 ± 0.2 ^a^	1.6 ± 0.2 ^a^
NLR (%)	Placebo	2.6 ± 0.2 ^a^	2.7 ± 0.3 ^a^	1.5 ± 0.1 ^a,^*
	RES-500	2.7 ± 0.3 ^a^	2.5 ± 0.2 ^a^	1.6 ± 0.2 ^a,^*
	RES-1000	2.8 ± 0.2 ^a^	2.6 ± 0.2 ^a^	1.6 ± 0.2 ^a,^*

Data are presented as mean ± SEM. Same superscript letters (a) indicate no significant differences among groups at *p* > 0.05, and baseline is compared with post-2 and -24 h, respectively. Administration effects were statistically analyzed with a paired Student’s *t*-test, compared with pre, * *p* < 0.05. RES, resveratrol; WBC, white blood cell; NLR, neutrophil/lymphocyte ratio.

## Data Availability

The data presented in this study are available on request from the corresponding author. The data are not publicly available due to subject’s privacy and confidentiality.

## Supplementary Materials

The following are available online at https://www.mdpi.com/article/10.3390/nu13093217/s1, Table S1. Participant characteristics and food intake at baseline; Table S2. The effect of RES supplementation on the blood biochemistry markers.
