# Supplementary material for: Protective and Recovery Effects of Resveratrol Supplementation on Exercise Performance and Muscle Damage following Acute Plyometric Exercise
Source: Nutrients. 2021 Sep 16;13(9):3217. doi: 10.3390/nu13093217 (PMC8469037; doi:10.3390/nu13093217)
Supplement: Supplementary file 1 [file nutrients-13-03217-s001.zip › nutrients-1338311-supplementary.pdf]

Table S1. Participant characteristics and food intake at baseline.

Characteristics	Placebo n=12	RES-500 n=12	RES-1000 n=12
**Age** (years)	21.3 ± 0.4[a]	21.2 ± 0.4[a]	20.7 ± 0.3[a]
**Height** (cm)	172.8 ± 1.6[a]	172.5 ± 1.3[a]	174.3 ± 2.4[a]
**Weight** (kg)	68.7 ± 1.8[a]	70.6± 2.1[a]	70.5 ± 2.9[a]
**BMI** (kg/cm^2)	23.0 ± 0.3[a]	23.7 ± 0.6[a]	23.2 ± 0.7[a]
**Muscle mass** (kg)	33.1 ± 1.0[a]	34.3 ± 1.0[a]	34.1 ± 1.3[a]
**Fat mass** (%)	15.1 ± 0.9[a]	14.7 ± 1.2[a]	14.9 ± 0.8[a]
**Carbohydrate** (g/day)	209 ± 10[a]	209 ± 12[a]	208 ± 9[a]
**Protein** (g/day)	79 ± 3[a]	76 ± 7[a]	79 ± 2[a]
**Fat** (g/day)	81 ± 4[a]	81 ± 9[a]	82 ± 4[a]
**Total calories** (kcal/day)	1882 ± 56[a]	1870 ± 79[a]	1885 ± 52[a]

Data are presented as mean ± SEM. BMI, body mass index.

Table S2. The effect of RES supplementation on the blood biochemistry markers.

Parameters	Group	Baseline	Pre	2h	24h	48h	72h
**AST** (U/L)	**Placebo**	21 ± 1 [a]	19 ± 1 [a]	18 ± 1 [a]	23 ± 2 [a,*]	21 ± 2 [a,*]	19 ± 1 [a]
	**RES-500**	21 ± 1 [a]	19 ± 1 [a]	19 ± 1 [a]	23 ± 1 [a,*]	22 ± 1 [a,*]	20 ± 1 [a]
	**Res-1000**	22 ± 1 [a]	19 ± 2 [a]	20 ± 2 [a]	23 ± 2 [a,*]	21 ± 1 [a,*]	19 ± 1 [a]
**ALT** (U/L)	**Placebo**	15 ± 1 [a]	19 ± 2 [a]	22 ± 2 [a,*]	21 ± 1 [a]	20 ± 1 [a]	22 ± 2 [a,*]
	**RES-500**	16 ± 1 [a]	18 ± 2 [a]	22 ± 2 [a,*]	21 ± 2 [a,*]	19 ± 1 [a]	23 ± 2 [a,*]
	**Res-1000**	15 ± 1 [a]	18 ± 1 [a]	22 ± 1 [a,*]	22 ± 2 [a,*]	19 ± 3 [a]	22 ± 2 [a,*]
**BUN** (mg/dL)	**Placebo**	17.7 ± 1.0 [a]	14.6 ± 0.8 [a]	13.2 ± 0.8 [a,*]	11.9 ± 1.0 [a,*]	15.3 ± 1.6 [a]	14.0 ± 1.1 [a]
	**RES-500**	17.2 ± 0.7 [a]	14.2 ± 0.9 [a]	13.2 ± 1.0 [a,*]	11.8 ± 1.1 [a,*]	14.7 ± 1.1 [a]	14.0 ± 0.5 [a]
	**Res-1000**	17.4 ± 1.4 [a]	14.7 ± 0.7 [a]	13.5 ± 1.4 [a]	11.0 ± 0.7 [a,*]	15.7 ± 0.8 [a,*]	14.6 ± 1.0 [a]
**CREA** (mg/dL)	**Placebo**	1.20 ± 0.02 [a]	1.26 ± 0.02 [a]	1.31 ± 0.02 [a,*]	1.25 ± 0.02 [a]	1.23 ± 0.02 [a,*]	1.23 ± 0.02 [a,*]
	**RES-500**	1.20 ± 0.02 [a]	1.26 ± 0.02 [a]	1.31 ± 0.02 [a,*]	1.25 ± 0.02 [a]	1.23 ± 0.02 [a,*]	1.24 ± 0.03 [a]
	**Res-1000**	1.19 ± 0.03 [a]	1.27 ± 0.03 [a]	1.31 ± 0.03 [a,*]	1.25 ± 0.02 [a]	1.23 ± 0.03 [a,*]	1.23 ± 0.02 [a,*]

Data are presented as mean ± SEM. Different superscript letters (a) indicate significant difference between groups at $p < 0.05$, and pre- is compared with post-2h, 24, 48 and 72h, respectively. Administration effects were statistically analyzed with a paired Student's *t*-test, compared with pre, * $p < 0.05$. AST, aspartate aminotransferase; ALT, alanine aminotransferase; BUN, blood urea nitrogen; CREA, creatine.
